# A novel music-based real-time fMRI neurofeedback interface modulates interhemispheric connectivity and enhances mood

**DOI:** 10.3389/fpsyt.2026.1757213

**Published:** 2026-04-30

**Authors:** Alexandre Sayal, João Pereira, Bruno Direito, Teresa Sousa, Miguel Castelo-Branco

**Affiliations:** 1 Coimbra Institute for Biomedical Imaging and Translational Research (CIBIT), University of Coimbra, Coimbra, Portugal; 2 University of Minho, Intelligent Systems Associate Laboratory (LASI), Guimarães, Portugal; 3 Department of Cognitive Neuroscience, Maastricht Brain Imaging Center, Maastricht University, Maastricht, Netherlands; 4 Centre for Informatics and Systems (CISUC), University of Coimbra, Coimbra, Portugal; 5 Institute of Physiology, Faculty of Medicine, University of Coimbra, Coimbra, Portugal; 6 Institute of Nuclear Sciences Applied to Health (ICNAS), University of Coimbra, Coimbra, Portugal

**Keywords:** immersive, interface, music, neurofeedback, reward

## Abstract

**Introduction:**

Music is a universal language that transcends cultures and is deeply rooted in human evolutionary history. Its creation and appreciation recruit the limbic and reward systems, leading to the evocation of emotions ranging from happiness and sadness to tenderness and grief. Here, we investigate the potential of music as an interventional tool in a novel neurofeedback connectivity-based experiment.

**Methods:**

This study proposes a musical interface for real-time functional magnetic resonance imaging neurofeedback that is adaptable to diverse experimental paradigms, namely the ones aiming at improving mood and other affective dimensions. Using a previously developed motor imagery connectivity-based approach, we evaluate its feasibility and efficacy by comparing the modulation of bilateral premotor cortex activity during functional runs with real versus sham (random) feedback in 22 healthy adults. We also assess its performance against a visual feedback interface. The experiment involves a 50-minute MRI session, including anatomical scans, a premotor cortex functional localizer run, and four neurofeedback runs (two with active feedback and two with sham feedback). Pre- and post-session questionnaires assess the neurobehavioral impact on mood, musical background (as a potential predictor of neurofeedback success), and subjective feedback experiences. During neurofeedback, participants perform motor imagery of finger-tapping, with feedback delivered as a dynamic, pre-validated chord progression that evolves or regresses based on the functional connectivity between left and right premotor cortex.

**Results:**

We found that our implementation of music-based feedback was successful, with participants managing to modulate their own connectivity using the proposed interface. The modulation performance was similar for active and sham runs, possibly due to the power of music to boost neuromodulation, but the network recruitment was stronger for active neurofeedback, including in the insula, putamen, and target regions of interest. Behaviorally, we found a decrease in tension and an improvement in the overall mood of the participants after the session.

**Discussion:**

When comparing our results to previous neurofeedback data with a visual interface, we found stronger brain activations, in particular in neurofeedback-relevant regions such as the insula and the putamen. This work shows that it is possible to directly modulate interhemispheric connectivity using a real-time functional magnetic resonance imaging musical interface with potential effects on mood and recruitment of saliency and learning networks.

## 1 Introduction

Neurofeedback (NF) is an innovative brain-computer interface (BCI) technique, enabling individuals to modulate their brain activity through real-time feedback. With applications ranging from cognitive enhancement to clinical therapy, NF holds significant potential for improving mental health and well-being in psychiatric and neurological conditions (1–3). However, advancing NF methodologies to optimize usability and effectiveness in real-world therapeutic settings remains a critical challenge for researchers (4).

The wide adoption of NF interventions is hindered by several challenges. A significant barrier is the high proportion of non-responders - individuals who fail to achieve consistent modulation of their brain activity - resulting in highly variable success rates across studies (5). Another challenge lies in the methodological heterogeneity of NF research, including inconsistencies in defining appropriate control groups for different study objectives (6) and the lack of standardized reporting for key success metrics (7). Addressing these issues is crucial for improving NF’s reliability and scalability in clinical and real-world settings.

**Figure 1:**
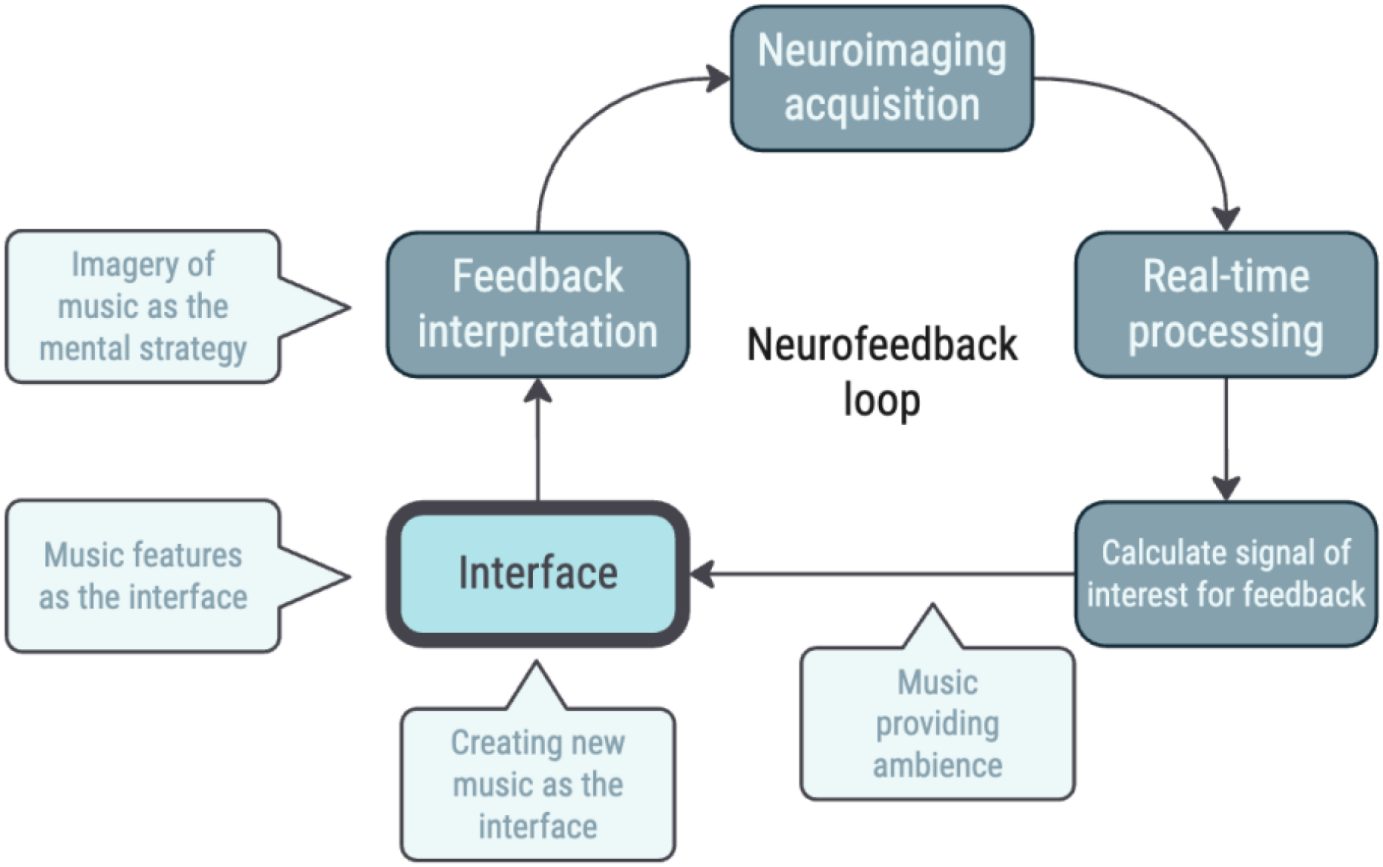
Illustration of the neurofeedback loop. The participant’s brain activity is acquired, analyzed, and transformed into feedback via an interface, which the participant uses to adjust their neural activity in real time. Music can play a role at various points in this process, from being the feedback to influencing participant engagement and interpretation. Here, we focus on the interface. Adapted from (8) under Creative Commons CC-BY license, v. 4.0.

Among the many factors influencing NF success, the design of the feedback interface - the medium through which neuronal information is conveyed to participants - plays a critical role (**Figure 1**). The type and presentation of feedback can strongly affect how participants engage with the task, influencing both the clarity of the signal and the subjective experience of control and immersion (9–11). Optimizing the interface design, using neuroscience-oriented approaches, may therefore help reduce the proportion of non-responders by enhancing participant engagement and the effectiveness of the feedback loop. We raised the hypothesis that using music as a strong modulator of emotion and reward circuits, one might achieve a higher likelihood of neuromodulation success. In this study, we aimed to design and validate a real-time functional magnetic resonance imaging (fMRI) NF approach based on modulation of functional connectivity using music as the interface between participants and their own brain activity - a step towards impacting affective processing and mood.

Music is a universal phenomenon, deeply rooted in human biology, culture, and history, with the ability to evoke and regulate emotions across contexts (12). Listening to music consistently engages the brain’s limbic and reward systems, enabling it to elicit both basic emotions, such as happiness and sadness, and complex ones, like tenderness or grief. In fact, the sound encoding mechanisms recruited during music listening and interpretation are based on a large and hierarchical network of brain areas, including primary and secondary auditory cortices, anterior cingulate cortex, insula, hippocampus, somatosensory cortex, and basal ganglia (13–16), regions tightly linked to the emotion circuits (17).

As a tool for emotion regulation, music allows individuals to modulate their emotional states by either suppressing undesired feelings or inducing new ones (18). Moreover, extensive evidence links music listening and training to enhanced brain plasticity, highlighting its potential as an effective medium for NF interventions (19–21).

Prior studies have explored the use of music in the context of NF approaches, and we recently conducted a systematic review to assess these effects (8). We investigated the primary motivations for incorporating music, the methodological approaches employed, and the reported outcomes. Many studies emphasized music’s ability to engage neural circuits related to emotion and reward, but causal explanations of these effects were still lacking. Moreover, no consensus was found on the imaging or behavioral metrics used to define NF success when music was involved. Focusing on whole-brain neural correlates of music stimuli and their interactions with target brain networks and reward mechanisms when designing music-based NF interventions can help to address these gaps. Recent efforts to decode brain patterns associated with interpreting valence and arousal (22–24) or complex emotions (15) in music add to our understanding of the underlying neural mechanisms, providing insights for the design of new music-based feedback approaches.

In this study, we developed a sham-controlled NF experiment with a novel interface based on musical chord progressions and implemented it in a real-time fMRI setup. Building on a previous NF experiment by our group, which established the feasibility of connectivity-based feedback during a motor imagery task (25) using a visual thermometer interface, we created a novel setup to explore music as the feedback medium. Our primary goal was to assess the feasibility of using connectivity and music-based feedback for real-time fMRI NF interventions. To this end, we investigated the following research questions:

1. Can participants successfully modulate both the activity and interhemispheric correlation of the bilaterial premotor cortex (PMC) using music-based feedback?
2. Does the music-based NF session positively affect participants’ mood?
3. Do participants accurately identify active NF runs (with contingent feedback) as more effective in terms of performance and feedback contingency compared to sham runs (with random feedback)?
4. Does contingent feedback elicit greater activation in reward- and learning-related brain regions compared to random feedback?
5. Aiming for a more immersive interface and a neuroscience-oriented approach, are the brain activation metrics stronger for the music feedback than for other approaches, such as the ones based on visual interfaces?

## 2 Methods

### 2.1 Study design overview

**Figure 2:**
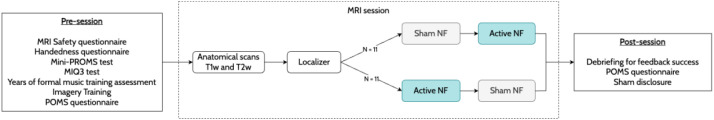
The experimental design includes a magnetic resonance imaging (MRI) session encompassing anatomical scans, a localizer run for the PMC, and four music-based neurofeedback (NF) runs (two with active feedback and two with sham). Participants completed pre- and post-session questionnaires to assess MRI safety, musical training, mood, and their subjective experiences regarding the feedback contingency.

The study design is summarized in **Figure 2**. We followed the CRED-nf (Consensus on the Reporting and Experimental Design of clinical and cognitive-behavioural neurofeedback studies) guidelines (7) to ensure transparent and rigorous reporting of our NF protocol; the completed checklist is provided in the **Supplementary Materials**.

After recruitment and before the Magnetic Resonance Imaging (MRI) acquisition session, participants completed a series of questionnaires. The first questionnaire assessed each participant’s compatibility with the MRI environment to ensure safety and compliance. Next, we characterized the sample based on three parameters: musical training, handedness, and motor imagery ability. Participants completed the Mini Profile of Music Perception Skills (Mini-PROMS) questionnaire (26), which assessed musical ability, and provided the number of years of formal music training. To assess handedness, we used the Edinburgh Handedness Inventory (EHI) questionnaire (27) variant based on (28). To evaluate motor imagery ability, participants completed the Movement Imagery Questionnaire-3 (MIQ-3) questionnaire (29, 30), which screens for visual and kinesthetic imagery capabilities.

Participants then watched a video explaining the NF experiment. This video detailed the number and type of sequences, the paradigm of the two tasks, and included a guided opportunity to practice motor imagery while listening to feedback music. To assess the impact of the NF session on mood, participants completed the Profile of Mood States (POMS) questionnaire (31) both before and after the MRI session. The scoring was obtained for six subscales: tension, depression, hostility, fatigue, confusion, and vigor.

The MRI session lasted approximately 50 minutes and included the acquisition of two anatomical scans, a functional localizer run, and four NF runs (two active and two sham). A crossover design was implemented: half of the participants received sham feedback during the first two runs, while the other half received sham feedback during the last two runs. The feedback shown during the sham NF runs was randomly generated.

After the MRI session, participants were asked to describe their experiences, including their perceived performance and feedback contingency during the NF runs. They were also asked to identify the best and worst runs regarding these parameters. Finally, the runs in which they received sham feedback were disclosed.

### 2.2 Participants

A sample of 22 healthy participants (13 females, mean age 33 ± 6 years, range 24–42 years) was recruited for this study. The research plan adheres to the legal regulations of the General Data Protection Regulation (GDPR), national legislation, and the Declaration of Helsinki. Furthermore, the study protocol and Informed Consent were approved in 2021 by the Ethics Committee of the Faculty of Medicine of the University of Coimbra (CE-060/2021). Based on previous research on neurofeedback (32), we aimed for an effect size of 1.09 (α = 0.05) for a modulation activity difference between active and sham conditions. Based on G*Power (33), the estimated sample size was 19 participants.

Participants had an average mini-PROMS score of 19.3 ± 5.6 (range: 11.0–31.5), indicating their level of musical ability. Motor imagery ability scores were 5.8 ± 0.7 for internal visual imagery (range: 4.3–7.0), 5.6 ± 1.1 for external visual imagery (range: 3.0–7.0), and 5.4 ± 0.9 for kinesthetic imagery (range: 4.0–7.0). All participants were right-handed, with an average laterality index of 83.8 ± 13.6 (range: 45.0–100.0).

### 2.3 Paradigms

**Figure 3:**
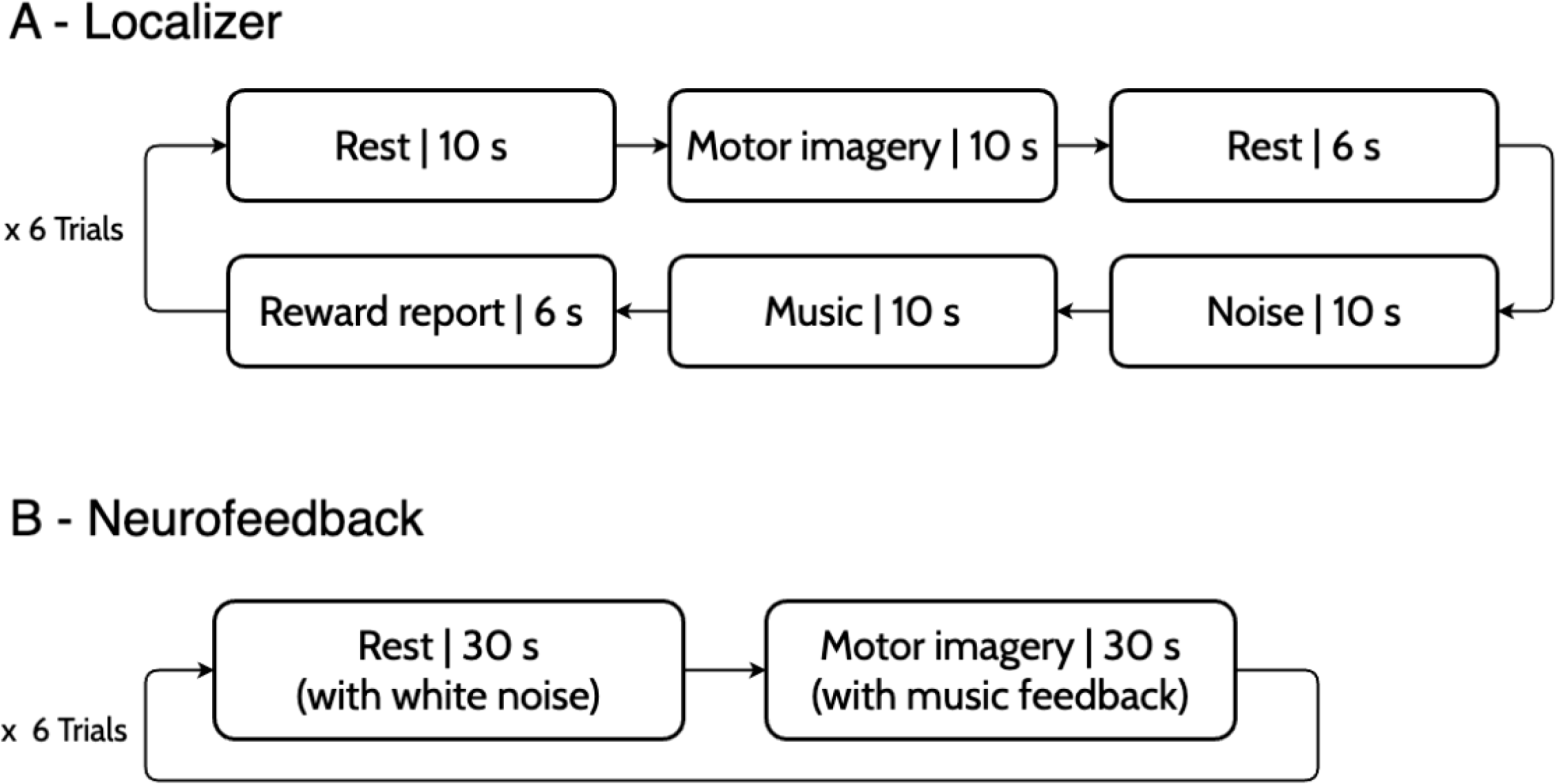
Functional paradigms for the localizer **(A)** and neurofeedback **(B)** runs. The localizer comprised 6 trials of interleaved blocks of rest, motor imagery, music listening, and music pleasantness report. During the neurofeedback runs, participants trained to control their PMC’s interhemispheric connectivity during the ‘Motor imagery’ blocks while imagining their hands and fingers moving. The feedback, presented only during these blocks, is based on a pre-validated rewarding chord progression that evolves or regresses according to the windowed correlation between the activities of the left and right PMC.

The main objective of the functional localizer was to identify the brain networks of motor imagery and music interpretation. The trial design is presented in **Figure 3A**, comprised of 6 trials, each containing 6 seconds of ‘Rest’, 10 seconds of ‘Motor imagery’, ‘Music’, and ‘Noise’, and 6 seconds of ‘Reward report’ conditions, and with a total duration of approximately 7 minutes. This run was used to identify the PMC on the left and right hemispheres, which are the target regions for the NF imagery runs. The participants were asked to imagine tapping the fingers of their hands during the ‘Motor imagery’ condition and to attentively listen to the music during the ‘Music’ condition. Afterward, they were asked to report the pleasantness of the music in five levels (unpleasant, slightly unpleasant, neutral, slightly pleasant, pleasant). The music played was sampled from the chords that were used in the imagery runs as feedback. In this run, the instructions and rating scale appeared on the screen.

The main objective of the NF runs was to train the participants in modulating the interhemispheric connectivity of the PMC, our imaging success metric, through the imagery of motor actions. The trial design is presented in **Figure 3B**. The ‘Motor imagery’ was accompanied by musical feedback, which was calculated using the windowed correlation (8-second window) between the activity of the left and right PMC. Participants were asked to imagine moving their hands and fingers (without actually moving) while listening to the music feedback. The instruction was auditory - the ‘Rest’ condition was indicated by white noise, and the ‘Motor imagery’ condition by the music feedback. Following the task of (25), halfway through the 30-second ‘Motor imagery’ block, a ‘beep’ sound was played - this indicated that participants should slowly decrease the frequency of the imagined movements until stopping close to the end of the block. This allowed for a prolonged variation of the BOLD activity in both premotor cortices, which would lead to a prolonged high correlation throughout the full block.

### 2.4 Neurofeedback setup

#### 2.4.1 Feedback music

**Figure 4:**
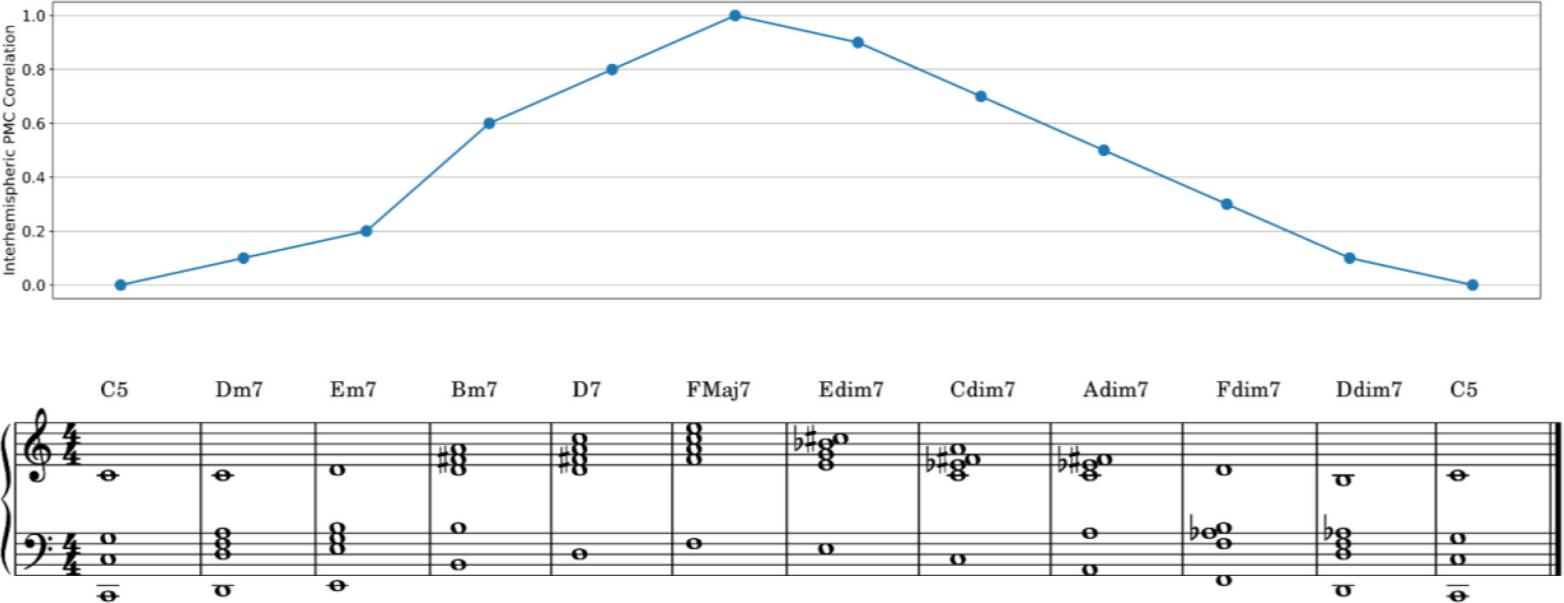
Feedback music - correspondence between an example interhemispheric premotor cortex (PMC) correlation time course and its translation into musical chords/feedback presented. Positive feedback is linked to higher base notes (chord tonics) and more pleasant chords (major 7th, minor 7th), while negative feedback is linked to lower base notes and less pleasant chords (diminished 7th).

In our approach, we mapped the correlation values to a harmonic progression where each 0.1 increment in correlation corresponded to a specific base note (i.e., tonic of the chord), allowing for ten possible base notes. The harmonic progression was dynamically shaped by these base notes, with the level of correlation (ranging from 0 to 1) determining the tonal foundation. Furthermore, the chord type was influenced by correlation changes (target metric) over time: when the correlation increased compared to the previous time window, the chord was either major 7th or minor 7th (consonant), whereas when the correlation decreased, a diminished 7th chord (dissonant) was played. This real-time mapping ensured that musical pleasantness, and hence positive or negative feedback, fluctuated in alignment with the correlation variation. In **Figure 4**, we illustrate a chord progression derived from a correlation time course, showing how each time point was associated with the chord the participant heard. The resulting harmonic shifts reflect the programmed relationship between correlation and musical feedback. We provide the audio of this example as **Supplementary Material**.

#### 2.4.2 Setup at the MRI

**Figure 5:**
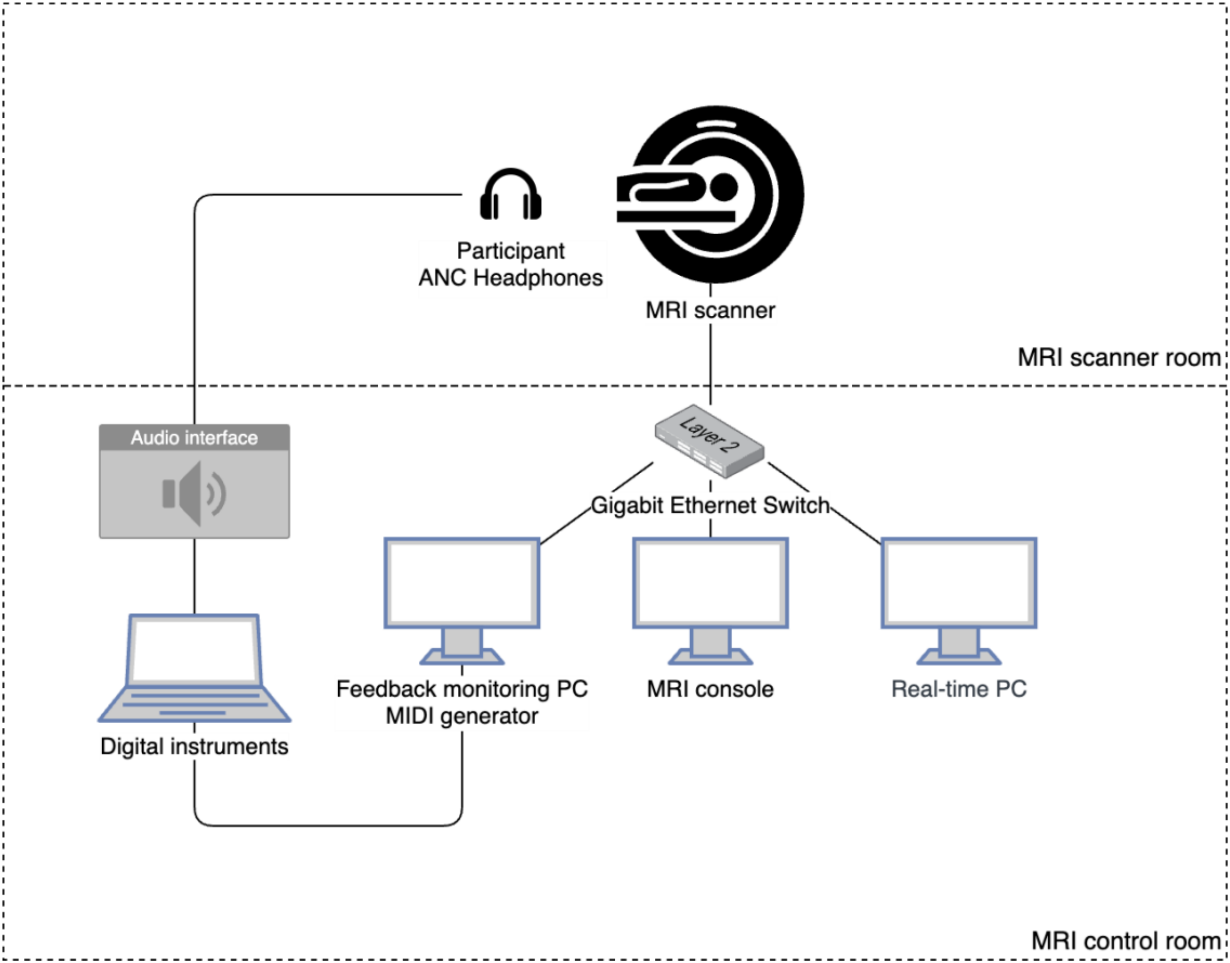
Schematic diagram of the real-time fMRI setup used to deliver music-based neurofeedback. The system enables real-time computation of interhemispheric correlation from fMRI signals and its translation into auditory feedback via MIDI generation. Functional images are acquired by the MRI scanner and processed by a real-time PC, with results transmitted over a local network to the feedback monitoring PC. This PC maps correlation values to musical parameters, generating MIDI signals that are converted to sound by digital instruments and played to the participant through active noise-cancelling (ANC) headphones.

The technical NF loop used during the MRI session is illustrated in **Figure 5**. The MRI console exported DICOM files in real-time, i.e., as they were acquired, using a proprietary add-in that transmitted the data to the real-time processing computer. This computer ran Turbo-BrainVoyager v4 software for real-time fMRI analysis, where the target regions of interest (ROIs) were previously defined in the bilateral PMC of each participant based on anatomical landmarks and functional activation observed during the localizer run (‘Motor imagery’ vs. ‘Rest’). Inter-run alignment was activated, which ensured the ROIs stayed valid during the entire session. A separate feedback monitoring computer ran a custom Python script that either received the windowed correlation values via TCP/IP for the real NF runs or generated random feedback for the sham NF runs, converted these values into a scale, and generated the MIDI signals which were sent to virtual instruments created in Logic Pro v11.1.2. The resulting audio feedback was delivered to the participants, updated every repetition time (TR), through the noise-canceling headphones.

#### 2.4.3 Real-time analysis

Real-time analysis was performed in Turbo-BrainVoyager v4. After the localizer run, the ROIs in the bilateral PMC of each participant were defined based on the activation map of the contrast between ‘Motor imagery’ and ‘Rest’ conditions. The statistical threshold was set to t-value > 3.1 (p < 0.001 uncorrected). Anatomical landmarks of the precentral gyrus, visible when superimposing the anatomical T1w image, were also considered in this definition. For all functional runs, the preprocessing steps included motion correction, inter-run alignment, and detrending. An iterative General Linear Model (GLM) was used to estimate the activation maps for each run. The design matrices included predictors for each of the experimental conditions and the six motion parameters (translation and rotation in the X, Y, and Z axes) as confound predictors.

### 2.5 MRI acquisition

The MRI acquisition was performed with a 3 T Siemens Magnetom Prisma fit scanner with a 64-channel head coil at the Institute of Nuclear Sciences Applied to Health from the University of Coimbra. Auditory stimuli were presented using MRI-compatible headphones (Optoacoustics Optoactive II). The scanning session started with the acquisition of two anatomical images: one 3D anatomical magnetization-prepared rapid acquisition gradient echo pulse sequence TR = 2530 ms, echo time (TE) = 3.5 ms, flip angle (FA) = 7°, 176 slices, voxel size 1.0 × 1.0 × 1.0 mm, field of view (FOV) = 256 × 256 mm) and one T2 space sequence (TR = 3200 ms, TE = 410 ms, 176 slices, voxel size 1.0 × 1.0 × 1.0 mm, FOV = 256 mm). A total of five functional runs were acquired using a 2D gradient-echo echo-planar imaging sequence (TR = 1500 ms, TE = 30 ms, flip angle = 75°, 26 slices, voxel size = 3.0 × 3.0 × 3.5 mm, slice gap = 0.5 mm, FOV = 210 mm, GRAPPA factor 2, echo spacing = 0.67 ms, bandwidth = 1700 Hz/px). For unwarping, a gradient field mapping sequence was acquired before the functional scans (TR = 400 ms, TE1 = 4.92 ms, TE2 = 7.38 ms, flip angle = 60°, 26 slices, voxel size = 3.0 × 3.0 × 3.5 mm, slice gap = 0.5 mm, FOV = 210 mm, bandwidth = 600 Hz/px). The participants’ physiological signals (respiratory and cardiac) were recorded during the functional runs using the scanner’s Physiological Measurement Unit (PMU). The respiratory signal was recorded at 50 Hz using a respiratory cushion, and the cardiac cycle was recorded at 200 Hz using a pulse sensor.

### 2.6 Offline MRI analysis

The MRI data were organized according to the Brain Imaging Data Standard (BIDS), using a customizable conversion tool (34) for the DICOM images and custom scripts to associate the task events. Preprocessing was performed in fMRIPrep v24.0.1 (35, 36) and included slice time correction, motion correction, unwarping, and normalization to the MNI space. For a complete description of the fMRIPrep methods, please refer to the **Supplementary Materials**.

All subsequent analyses were performed using custom Python scripts based on the package Nilearn v0.10.4 (37, 38).

#### 2.6.1 Visual interface dataset processing

To compare our music-based interface with the visual interface used in (25), we obtained raw data previously acquired by the authors. The dataset was then converted to the BIDS format, preprocessed using the same version of fMRIPrep, and analyzed with an identical statistical approach based on GLMs, as the task is identical, only differing in the feedback modality. For a detailed description of the acquisition parameters and protocol, please refer to the original paper (25).

### 2.7 Statistical analysis

#### 2.7.1 Behavioral data

During the localizer run, participants reported how pleasant/unpleasant each chord was to listen to: they rated each chord on a 5-level scale from unpleasant to pleasant, using the available buttons inside the MRI. We aimed to confirm that the pleasant and unpleasant labels we defined were valid for our sample. After extracting the report values for each type of chord, we statistically compared pleasant and unpleasant chords using a Mann-Whitney U test (this non-parametric test was selected due to unpaired, non-normal samples and a not-very-large sample size).

Participants answered the POMS questionnaire immediately before and after the MRI neurofeedback session. The statistical comparison between the pre- and post-session POMS scores was performed using a paired-sample Wilcoxon signed-rank test (this non-parametric test was chosen due to paired, non-normal samples and a not-very-large sample size) with Holm-Bonferroni correction (p = 0.05).

We also look for correlations between the behavioral metrics (POMS, Mini-PROMS, MIQ-3) and the success metric (average interhemispheric correlation during active NF) using linear regression.

#### 2.7.2 Activation maps

For the first-level analyses, GLMs were employed to obtain activation maps for each of the contrasts of interest and participant. For the localizer run, we estimated the contrast ‘Music’ + ‘Motor imagery’ > ‘Noise’ + ‘Rest’, while for the NF runs, the contrast was ‘Motor imagery’ > ‘Rest’. The design matrices included predictors for each of the experimental conditions and confound predictors for head motion (six motion parameters + first-order derivatives + powers, a total of 24), the mean signals of the cerebro-spinal fluid (CSF) and white matter masks, and 20 regressors based on the physiological signals, including RETROICOR, respiratory volume per time and heart rate variability responses (RVT/HRV), estimated with PhysIO toolbox (39) - these last are critical when denoising data from connectivity-based NF experiments (40). We also applied temporal high-pass filtering with a cut-off frequency of 0.008 Hz and considered a second-order autoregressive model AR(2) as the temporal variance model. The second-level group analyses considered spatial smoothing with a Gaussian kernel of full-width at half maximum = 6 mm and were corrected for multiple comparisons depending on the task objective and imaging protocol: with False Discovery Rate (FDR) at q = 0.005 (localizer, single run per participant) or with Bonferroni’s method at p = 0.05 (NF runs, two per participant and feedback type). Additionally, we considered a minimum cluster size of 10 voxels.

#### 2.7.3 Comparing active and sham feedback

One of the questions of this feasibility study regards the activation patterns of contingent feedback vs. sham. For this comparison, we considered the thresholded group-level activation maps (p = 0.05 with Bonferroni’s correction, k > 10) of active and sham feedback runs and defined a binary mask including the significant voxels for each of them. Then, we summed these masks to obtain a map of overlap between the two conditions - in practice, in each voxel, we show if the activity was significant for both conditions, only for active, or only for sham feedback. This map provides an overview of the pattern of network recruitment, while we look more closely at the reward and learning circuits.

#### 2.7.4 Comparing music and visual interfaces

To search for differences in the activation maps retrieved from our music-based active NF runs and the visual interface experiment, we extracted the contrast maps between ‘Motor imagery’ and ‘Rest’ for all subjects (N = 22 for music and N = 10 for visual interfaces). Then, we employed a second-level, unpaired, two-sample t-test (two-sided) on the brain maps to see the effect of the interface difference across subjects (Music NF vs. Visual NF). This comparison was corrected with Bonferroni’s method at p = 0.05.

## 3 Results

### 3.1 Localizing the PMC for neurofeedback

**Figure 6:**
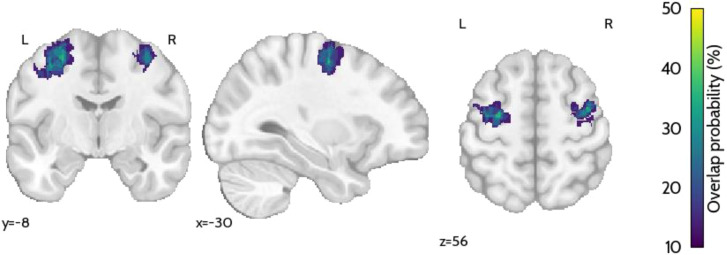
Brain probability map showing the percentage of overlap of the target ROIs (bilateral PMC) across participants.

The localizer run allowed for the functional definition of each participant’s left and right PMC, the targets for the neurofeedback runs, during the MRI session. This step was performed online based on the activation map contrasting the response to the ‘Motor imagery’ condition and the ‘Rest’ condition. The average MNI coordinates of the left PMC were (-36, -6, 54) and for the right PMC (37, -4, 55). The coordinates for each participant’s target regions are provided in **Supplementary Table 1**. The map showing the percentage of overlap of these regions across participants is presented in **Figure 6**.

**Figure 7:**
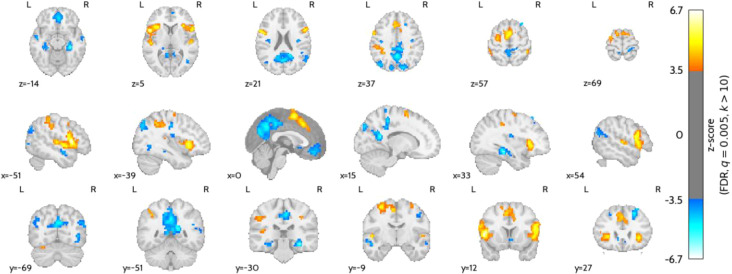
Brain activation map of the localizer run, contrasting motor imagery and music conditions vs. noise listening and rest. This map highlights regions linked to music perception (Helschl’s gyrus, temporal superior gyrus) and motor imagery (precentral gyrus, supplementary motor area).

Based on the localizer data, we look into the brain regions expected to be recruited during NF. In this test, we contrasted the activity both during motor imagery and music listening conditions against noise listening and rest conditions. The statistical map is shown in **Figure 7**. By performing clustering in this map, we identify significant clusters in the frontal inferior operculum, insula, precentral gyrus, SMA, temporal superior gyrus, Helschl’s gyrus, and cerebellum (clusters detailed in **Supplementary Table 2**). We also note the very clear deactivation of the default mode network, a marker of task engagement.

The behavioral data from the reports regarding how pleasant/unpleasant each chord was to listen to confirmed our association - the statistical comparison between the ratings of pleasant and unpleasant chords revealed a significant difference (**Supplementary Figure 3**).

### 3.2 Assessing the modulation of PMC interhemispheric correlation

**Figure 8:**
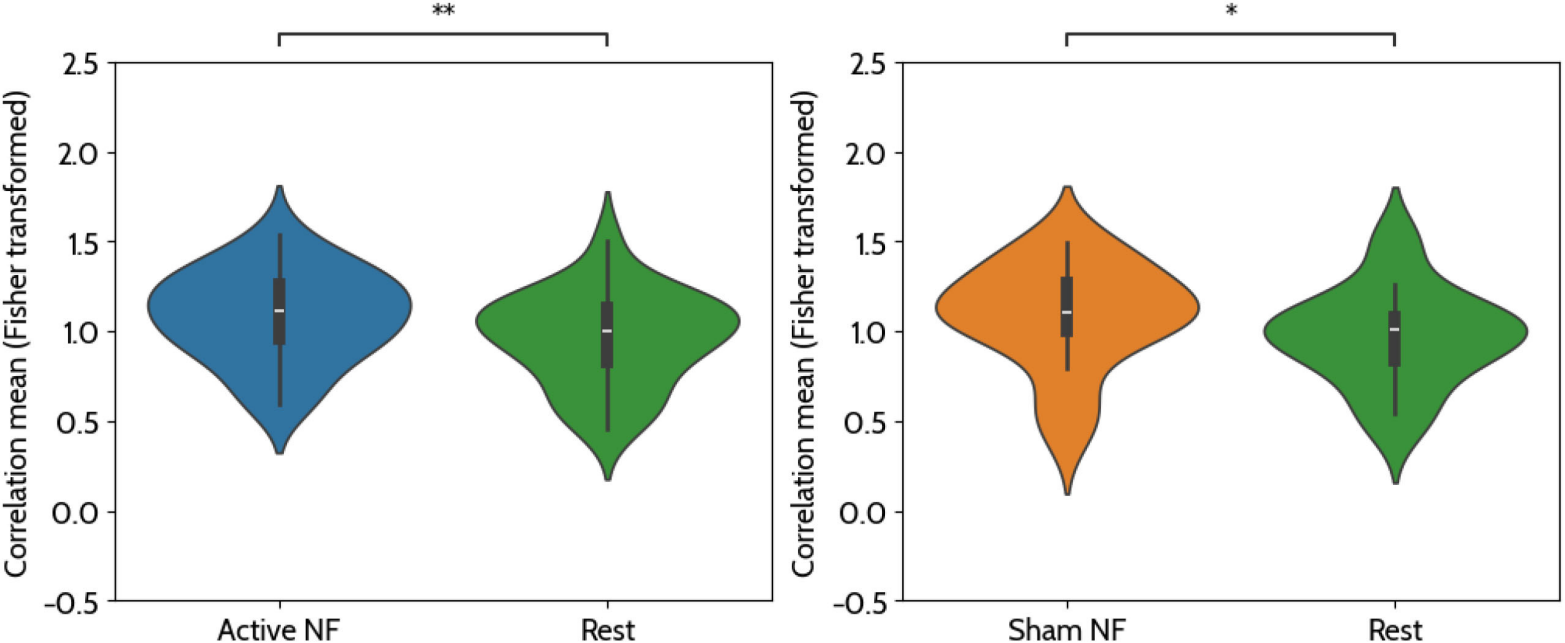
Average correlation values, as measured by the real-time software, during active neurofeedback (NF), sham NF, and rest conditions across subjects (Fisher transformed). A one-sided Wilcoxon signed-rank test was conducted to compare mean correlation values between the NF and ‘rest’ conditions. Results showed that correlation values were significantly higher in the ‘active NF’ condition (Mdn = 1.11) compared to the ‘rest’ condition (Mdn = 1.00), W = 215.00, p = 0.003 (one-tailed, corrected for two comparisons) and also significantly higher in the ‘sham NF’ condition (Mdn = 1.11) compared to the ‘rest’ condition (Mdn = 1.00), W = 201.00, p = 0.014 (one-tailed, corrected for two comparisons). * - 0.01 < p < 0.05 and * - ** 0.001 < p < 0.005.

**Figure 9:**
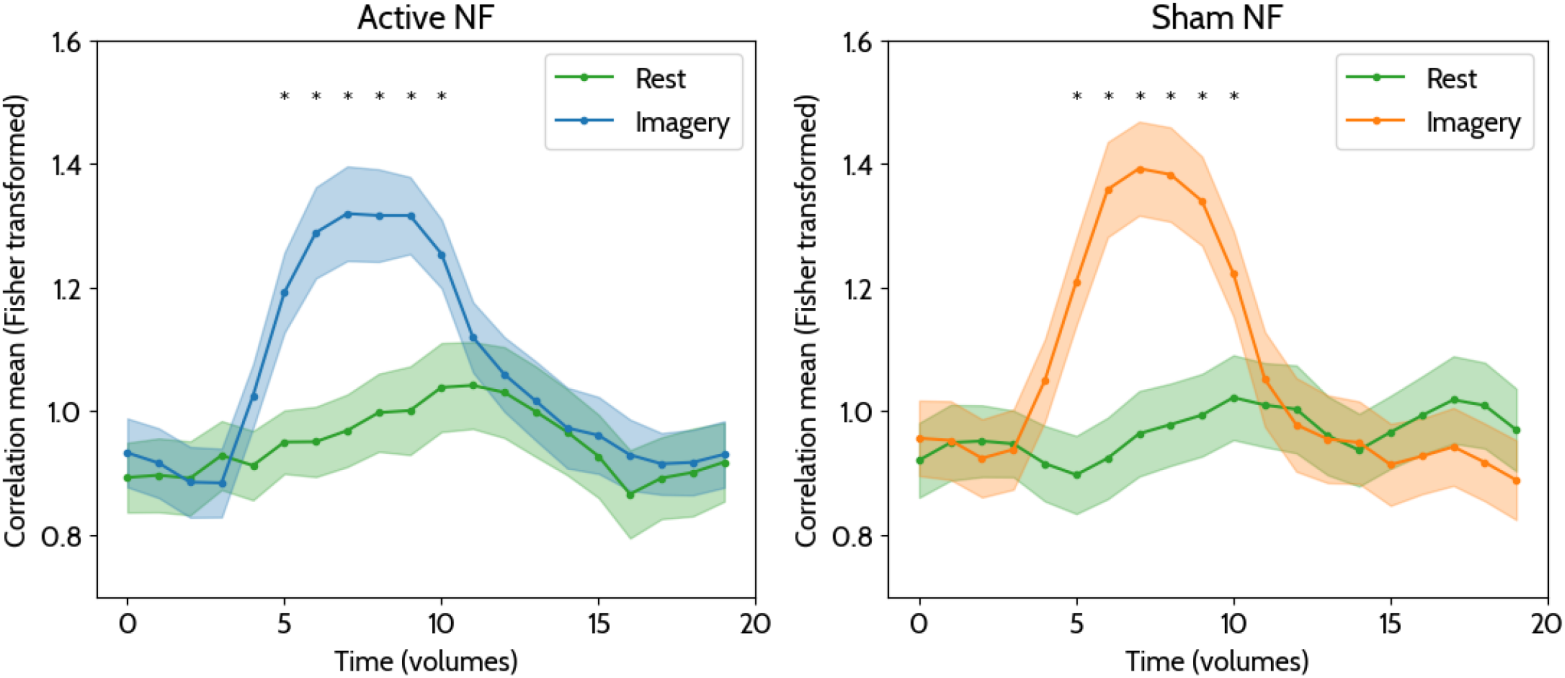
Average correlation time course during the ‘Motor imagery’ and ‘Rest’ conditions, as measured by the real-time software, for the active and sham neurofeedback (NF) runs. Each data point corresponds to the correlation in the sliding window that includes the 8 points that precede it. The correlation values were transformed to Fisher’s z. Paired Wilcoxon tests per timepoint between imagery and rest correlation values (* - p < 0.05, corrected for two comparisons).

The basis for the feedback provided, and also the modulation target for the participants, was Pearson’s correlation between the BOLD signals of the left and right PMC, as defined in the localizer run. These correlations were estimated in an 8-second sliding window across time during the acquisition. In **Figure 8**, we display the average of the estimated correlations across subjects for the active and sham runs during the NF ‘Motor imagery’ and ‘Rest’ blocks. In **Figure 9**, we display the average time course of such correlation in both NF conditions.

### 3.3 Participants’ mood before and after the NF session

**Figure 10:**
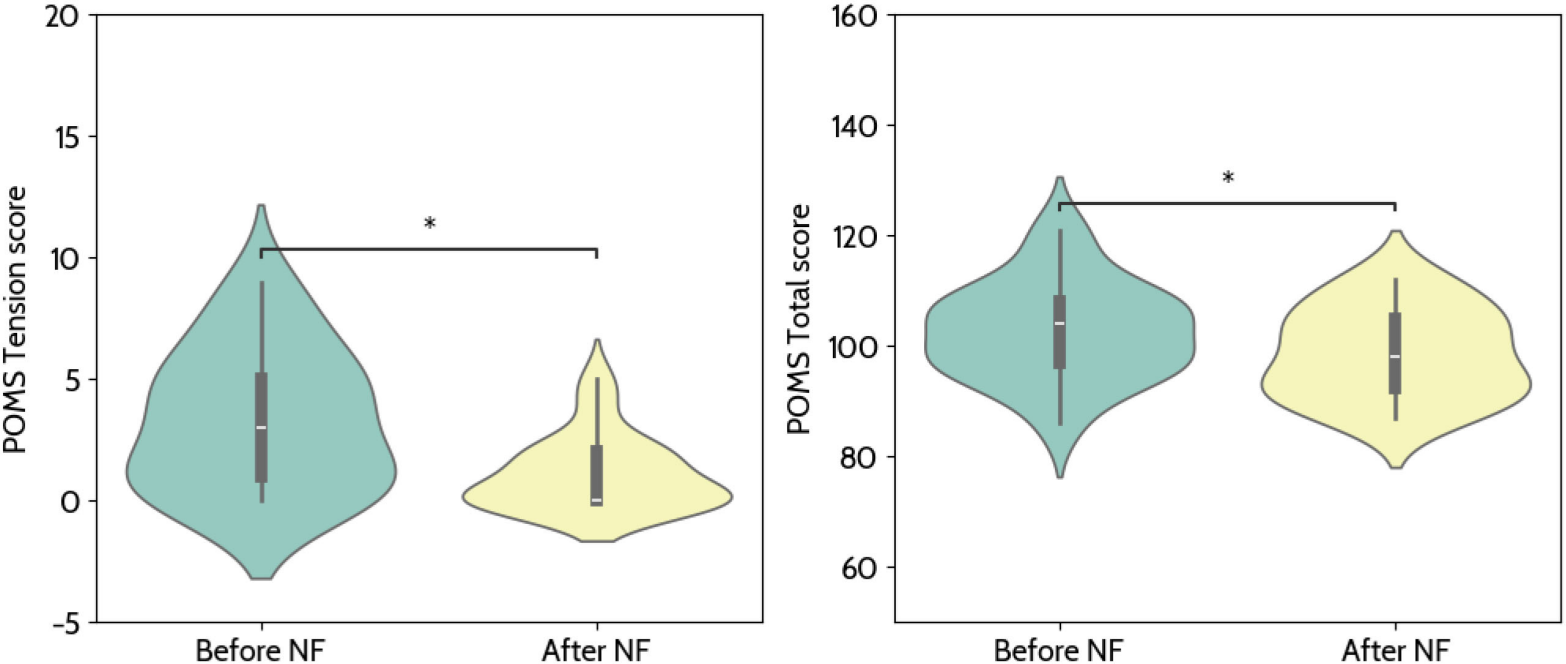
Comparison of the POMS scores before and after the neurofeedback (NF) session. We found a significant decrease in the tension subscale score and the total score with a paired sample Wilcoxon test after correcting for multiple comparisons with Bonferroni’s method (* - 0.01 < p < 0.05).

The assessment of participants’ mood before and after the MRI NF session was performed based on the self-reports of the POMS questionnaire. A significant decrease was found for the Tension sub-scale score (Mdn before = 3, Mdn after = 0, p = 0.012, W = 4.0, r = 0.924) and for the Total score (Mdn before = 104, Mdn after = 98, p = 0.012, W = 28.0, r = 0.673) - **Figure 10**. The results for the remaining sub-scales do not present a significant difference between time points and are presented in **Supplementary Figure 4**.

### 3.4 Debriefing participants after the NF session

After the scanning session, participants were debriefed regarding the overall experience of the NF session, if they were able to perform the task, and lastly, if they could identify which was the best and worst run out of the four, both regarding their performance and the feedback contingency. 17 out of 22 participants (77%) reported an Active feedback run as the best, and 15 out of 22 participants (68%) reported a Sham feedback run as the worst. Also, most participants reported that imagery during the second part of the block (while decreasing imagined movement frequency) was harder when compared to the first part.

### 3.5 Extracting brain activation patterns during neurofeedback

**Figure 11:**
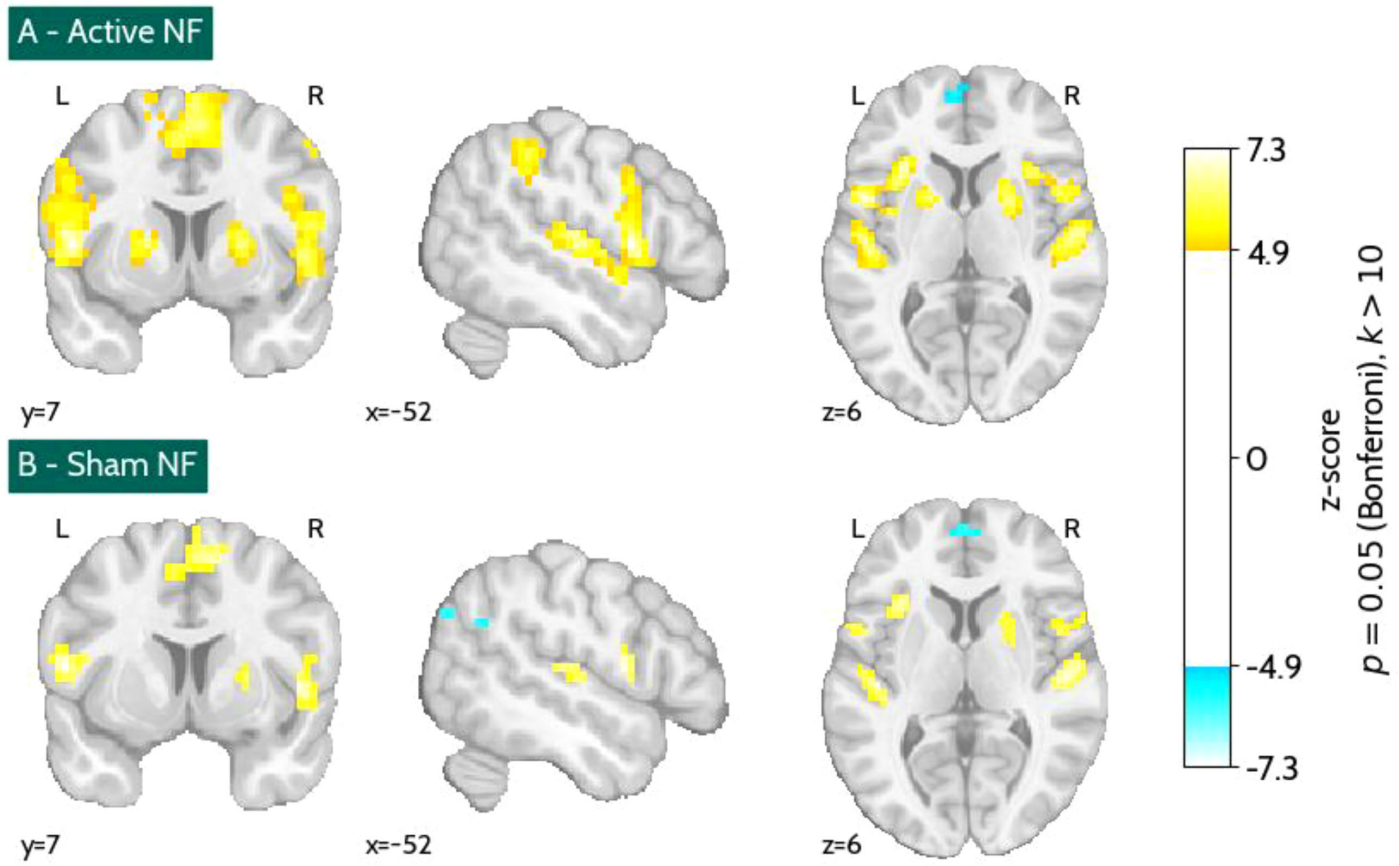
Brain activation maps of the active **(A)** and sham **(B)** neurofeedback (NF) runs contrasting ‘Motor imagery’ with ‘Rest’ (Bonferroni’s correction, p = 0.05, k > 10).

**Figure 11** presents the brain activation maps for the active (A) and sham (B) NF runs, showing regions with significant activation when contrasting ‘Motor imagery’ with ‘Rest’. The corresponding clustering analysis (detailed in **Supplementary Tables 3** and **4**) reveals activation in several cortical and subcortical regions involved in motor planning, sensorimotor integration, auditory processing, and cognitive-affective regulation. Key regions include the SMA, precentral gyrus, and inferior parietal lobule, which are associated with motor execution and spatial coordination. Additionally, consistent engagement of the insula, putamen, and inferior frontal gyrus suggests involvement of interoceptive networks. The superior temporal gyrus and Heschl’s gyrus were also recruited, reflecting the role of auditory processing in the NF task.

**Figure 12:**
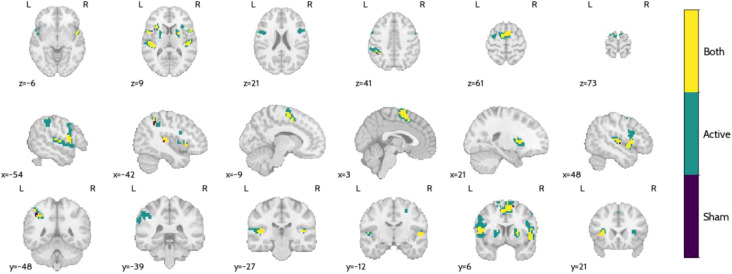
Comparison between the thresholded (Bonferroni’s correction, p = 0.05, k > 10) active and sham NF maps. Voxels for which both active and sham NF elicited significant activations are shown in yellow, while the ones for which only active NF elicited a significant activation are shown in green, and for sham-only in purple.

These regions were observed in both active and sham conditions, but with differential cluster peak activation and size. **Figure 12** presents a comparison between the thresholded activation maps for the active and sham NF conditions, highlighting regions that exhibited significant activation exclusively in one condition or both. **Table 1** provides a detailed breakdown of the clusters that were significantly active only in the active NF condition. These include regions such as the insula and putamen, precentral gyrus, SMA, inferior frontal gyrus, superior temporal gyrus, and inferior parietal lobule.

**Table 1:** Clustering of the active NF-only regions in **Figure 12**. For each cluster, we provide the coordinates in MNI space and the label of the AAL3 atlas.

Cluster ID	X	Y	Z	AAL3
1	-57	-27	10	Temporal_Sup_L
2	-51	-39	42	Parietal_Inf_L
3	-48	6	6	Frontal_Inf_Oper_L
4	-30	-6	58	Precentral_L
5	-30	24	2	Insula_L
6	-21	6	6	Putamen_L
7	-3	0	66	Supp_Motor_Area_L
8	9	6	66	Supp_Motor_Area_R
9	18	6	6	Undefined
10	27	-6	50	Precentral_R
11	36	18	6	Insula_R
12	48	9	22	Frontal_Inf_Oper_R
13	48	-24	14	Temporal_Sup_R
14	54	0	46	Precentral_R
15	54	3	2	Rolandic_Oper_R

### 3.6 Contrasting music and visual interfaces

**Figure 13:**
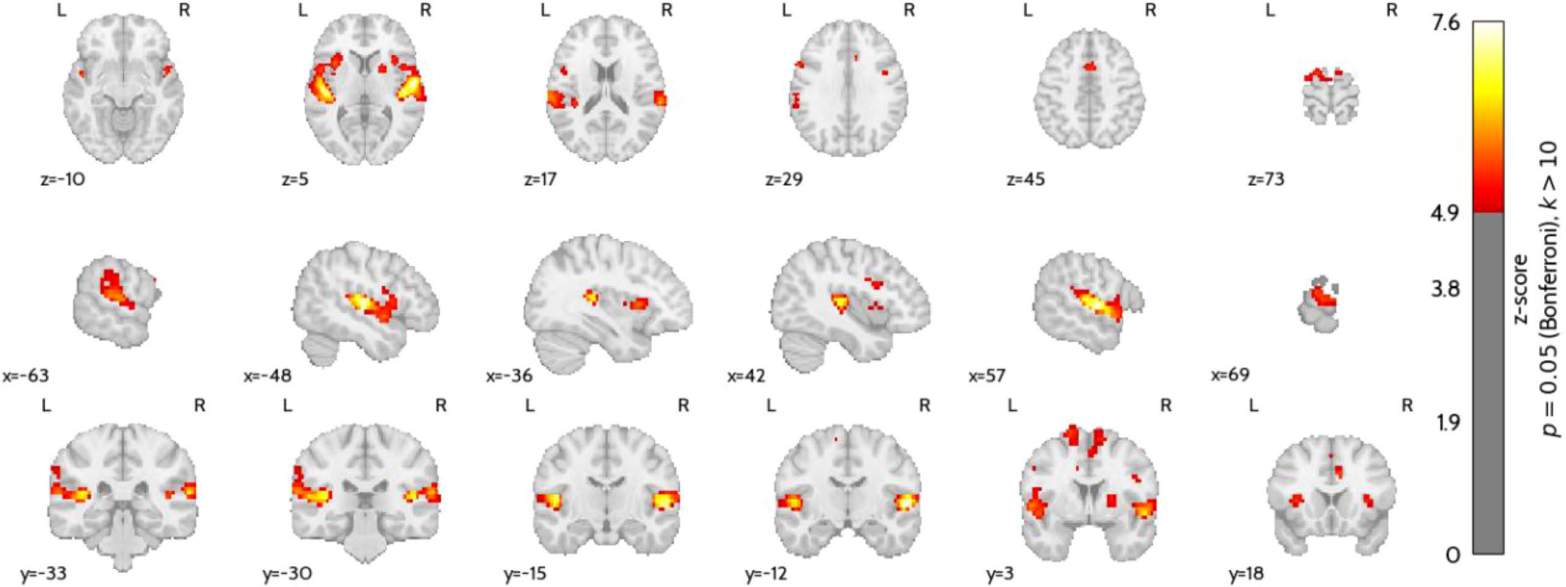
Brain map for the statistical contrast between the music-based active NF runs and the active NF runs with the visual interface of (25). In each study, the contrast considered was ‘Motor imagery’ vs. ‘Rest’ (Bonferroni’s correction, p = 0.05, k > 10).

**Figure 13** presents the second-level comparison between the brain activity patterns elicited during music-based active NF in the present study and those observed in the visual-based active NF in the study of (25). **Supplementary Table 5** details the clusters where significant differences were found (Music NF > Visual NF). Increased activation was observed in auditory-related regions, including Heschl’s gyrus, the superior temporal gyrus, and the Rolandic operculum, highlighting the engagement of auditory processing networks during music-based NF. Additionally, activation differences were identified in motor control-related areas, such as the SMA, precentral gyrus, insula, and putamen, suggesting potential differences in sensorimotor integration between the two feedback modalities. The mid-cingulate cortex and inferior frontal gyrus also showed differential activation, which may be linked to cognitive control and emotional processing.

## 4 Discussion

This study probed the use of music as a NF interface and investigated its capacity to modulate interhemispheric connectivity during motor imagery and the impact on affective measures such as mood. By comparing music-based feedback with both sham and visual NF conditions, we aimed to characterize the neural mechanisms engaged by different feedback modalities and assess the feasibility of using connectivity-based NF coupled with music as a neurobehavioral and impactful alternative to classical NF approaches.

### 4.1 Modulation of PMC interhemispheric connectivity

The primary objective of NF training in this study was to modulate interhemispheric connectivity in the PMC using music feedback. Importantly, the determination of whether the training was effective is a matter of debate, with currently no standardized method for measuring success (4). Our results indicate that the PMC connectivity modulation was successfully achieved, as the correlation during imagery blocks was stronger than during rest.

This result was particularly evident for the first half of the block, a modulation pattern which was also observed in (25). Sustaining correlation is not trivial, and actually requires skilled imagery, because as the BOLD signal reaches the plateau, correlation decreases to around zero - this is why we instructed participants to change the frequency of the imagined movement after the middle of the block. However, we did not verify the hypothesized decrease in BOLD after the ‘beep’ to keep a high correlation (**Supplementary Figure 1**), which consistently matches participants’ reports about the difficulty of decreasing imagery frequency.

### 4.2 The impact of music-based connectivity NF on behavioral measures and mood

Attentional factors seem to play a key role in both NF performance and learning, while motivation and mood have been identified as moderate predictors of success (5). Here, we assessed changes in mood as a target outcome measure via the POMS self-reporting questionnaire. We found that the overall score and the Tension subscale decreased after the NF session, which are consistent with signs of mood improvement. This scale has been found to change after NF in previous studies reporting a full NF intervention in autism spectrum disorder using a visual interface exploiting facial expressions (2) and in major depressive disorder using a visual thermometer (41, 42). In our case, none of the differences are correlated with the success metric (**Supplementary Figure 7**).

Following up on these results, performing a music-based NF task could have a direct clinical effect, since it provides the opportunity for emotion control. We recently reviewed how music is being used in NF loops (8), finding six studies out of the 15 reviewed also reporting significant changes in behavioral metrics (e.g., affective and mood-related measures, cognitive functioning scores, attention and mental control scores) after the NF intervention.

Regarding the Mini-PROMS and MIQ-3 questionnaires, we found no statistically significant correlations with the success metric (**Supplementary Figure 5, 6**). This means that, in this case, none of these would predict individual NF success (43) - the search for a outcome biomarker, not necessarily linked to the imaging, that could reflect the effects of reinforcement learning interventions (such as NF) is still a matter of debate (42).

### 4.3 Participants’ perception of contingency

The reported best and worst runs, as perceived by the participants, mostly match one of the active and sham runs, respectively. This reassures us that participants were able to perceive the presence of contingent, i.e., valid feedback. We note that the participants were unaware that sham feedback was going to be used in two of the NF runs. The utilization of sham feedback as the control condition for NF specificity is a matter of debate (4). The detection of non-contingent feedback, i.e., the loss of control, could lead to frustration or the abandoning of a valid strategy of neuromodulation. Anecdotally, some participants reported that after a sham run, in which the feedback did not work as expected, they tried harder to focus on the imagery task and make the feedback work - this is a possible confounding effect that might partially explain the results that follow.

### 4.4 Active vs. Sham feedback

In a sham-controlled study, the perception of whether the feedback signal aligns with effort may not differ significantly between groups (6). Many participants receiving real NF struggle to perceive a direct relationship between their effort and signal changes due to the inherent difficulty of self-regulating neural activity (44, 45). This disconnection between intention and perceived outcome contributes to a common experience of non-contingency, reported across both active and sham conditions (9, 46). If participants apply consistent effort, the relationship between effort and signal change is likely to be of similar magnitude in both groups. This perceived equivalence in feedback success may balance participants’ subjective experience, despite differences in signal origin.

Our results support this interpretation: we found no significant difference in the neural success metric when comparing active and sham feedback conditions. This outcome was anticipated, but we hypothesized that differences would emerge in the broader network dynamics, particularly in reward and learning-related circuits, namely the putamen (47). This hypothesis is supported by previous work suggesting that contingent feedback engages reinforcement learning processes, even in the absence of conscious performance evaluation (1, 48). Remarkably, we observed greater and more extensive activation in the putamen and insula during active NF. These regions have well-established roles in the context of NF: the putamen, as part of the basal ganglia, is involved in learning, reward prediction, and habit formation, while the insula is crucial for interoception, emotional salience, and monitoring internal states. Both are frequently reported in NF studies examining learning, motivation, and affective processing (48, 49). Their stronger engagement in the active condition suggests that, although the success metric alone may not distinguish between groups, the contingency of the feedback loop in the active NF condition recruits additional neural systems, potentially supporting the development of internal models of control and reward-based learning.

### 4.5 Music vs. visual interface for NF

Previous research has integrated music into NF paradigms, as reviewed in (8), yet important questions remain. One key aspect of these implementations is the understanding of the neural correlates of music-evoked emotions, which have been extensively characterized (14). Music engages brain regions involved in emotion processing and reward, such as the limbic system, auditory cortex, and basal ganglia, overlapping with the networks typically targeted by NF (48). This overlap suggests that music may not only serve as a feedback modality but may also potentiate NF effects by co-activating these functional systems. In our results comparing brain activity during music-based and visual NF, we observed stronger activation in music-related regions, including in the Heschl’s gyrus and SMA, but also in the putamen, insula, and the cingulate cortex (the latter also part of the salience network). These regions play distinct yet interconnected roles relevant to NF. Heschl’s gyrus, part of the primary auditory cortex, is crucial for processing complex sound features, and its activation indicates that participants were effectively perceiving and engaging with the musical feedback. The SMA is involved in motor planning and imagery, which aligns with the task demands and suggests successful task engagement. The putamen, a component of the basal ganglia, is implicated in motor learning and reward processing, and its activation may reflect reinforcement mechanisms facilitated by the emotionally salient and rewarding nature of music. Lastly, the cingulate cortex, particularly its mid and anterior regions, is associated with attention, cognitive control, and emotional regulation - all central to self-regulation and learning during NF. Its recruitment, together with the insula, suggests an important role for the saliency network. Nevertheless, these results should take into consideration the different sample sizes of the music and visual interface groups.

Compared to traditional visual NF interfaces, which provide explicit and structured feedback, music may offer a dynamic and emotionally engaging alternative. Music can act as a probe for emotional and reward-related processes, potentially enhancing NF learning through mechanisms such as emotional contagion (50). This interplay between emotion, reward, and self-regulation may offer unique advantages over purely visual feedback, making music-based NF a promising avenue for further research.

### 4.6 Future directions

In our study, we employed a harmonically simple but easy-to-understand chord progression as the interface to provide musical feedback of a motor imagery task. The base note reacted to the strength of connectivity, and what we defined as the ‘pleasantness’ of the chords to the direction of change in correlation. This pleasantness can be understood as a form of musical valence, a key dimension for triggering positive vs. negative feedback, which has well-documented neural correlates when encoded in music (22, 51–53).

Looking ahead, future studies could adopt a more naturalistic approach by integrating real music stimuli or more complex harmonic progressions. While this could enhance the ecological validity of music-based NF, it also introduces greater variability in how participants interpret the feedback. Balancing complexity and interpretability will be crucial in refining this approach for both experimental and clinical applications.

Regarding the crossover design employed in our study, while it was intended to balance the presentation of active and sham NF blocks and minimize order effects by equally distributing potential trends related to learning or fatigue across participants, we acknowledge that practice or fatigue confounds may still influence outcomes. Future studies should incorporate run order as a covariate in analyses to more rigorously control for these factors and enhance the interpretability of results.

Future studies may consider incorporating motor imagery chronometry as an additional metric to assess not only the capacity to generate motor imagery but also the ability to sustain it and the time required to initiate the process, which is particularly relevant for short NF blocks. This approach, when combined with self-report questionnaires such as the MIQ-3, can provide a more comprehensive evaluation of motor imagery ability, as findings suggest that chronometric and self-report measures address different components of imagery quality and should be used together (54). Such detailed characterization is fundamental for stratifying participants, identifying the best candidates/learners for NF strategies, and ultimately the optimizing protocol design and the therapeutic outcomes.

Beyond methodological advancements, we believe that the novel NF implementation presented here holds significant potential for clinical applications, particularly regarding how music can modulate brain mechanisms which are relevant for self-regulation. This seems especially relevant for emotion regulation interventions, given music’s recruitment of regions of the salience network (55), and in pain management, given the considerable overlap of the pain and music perception networks (56). The effects of music on pain perception and its reduction are well-documented (57), yet their clinical relevance remains underexplored (56, 58). Building on our previous work demonstrating the feasibility of NF in the context of pain empathy using a visual interface (59), future clinical trials integrating this music-based NF interface could offer a novel framework to investigate the mechanisms of music-induced analgesia and its neural correlates, potentially paving the way for innovative therapeutic interventions.

## Data Availability

All code used in this study is available in the GitHub repository (https://github.com/CIBIT-UC/musicnf-novelinterface). The dataset, formatted in BIDS, can be accessed at Zenodo (https://doi.org/10.5281/zenodo.14803374). This study was preregistered on OSF (https://doi.org/10.17605/OSF.IO/AHXNB).
